# Cell Senescence-Related Pathways Are Enriched in Breast Cancer Patients With Late Toxicity After Radiotherapy and Low Radiation-Induced Lymphocyte Apoptosis

**DOI:** 10.3389/fonc.2022.825703

**Published:** 2022-05-24

**Authors:** Ester Aguado-Flor, María J. Fuentes-Raspall, Ricardo Gonzalo, Carmen Alonso, Teresa Ramón y Cajal, David Fisas, Alejandro Seoane, Álex Sánchez-Pla, Jordi Giralt, Orland Díez, Sara Gutiérrez-Enríquez

**Affiliations:** ^1^Hereditary Cancer Genetics Group, Vall d’Hebron Institute of Oncology (VHIO), Vall d’Hebron Barcelona Hospital Campus, Barcelona, Spain; ^2^Radiation Oncology Department, Santa Creu i Sant Pau Hospital, Barcelona, Spain; ^3^Statistics and Bioinformatics Unit, Vall d’Hebron Institut de Recerca (VHIR), Vall d’Hebron Hospital Universitari, Vall d’Hebron Barcelona Hospital Campus, Barcelona, Spain; ^4^Medical Oncology Department, Santa Creu i Sant Pau Hospital, Barcelona, Spain; ^5^Medical Physics Department, Vall d’Hebron Hospital Universitari, Vall d’Hebron Barcelona Hospital Campus, Barcelona, Spain; ^6^Genetics, Microbiology and Statistics Department, Universitat de Barcelona, Barcelona, Spain; ^7^Radiation Oncology Department, Vall d’Hebron Hospital Universitari, Vall d’Hebron Barcelona Hospital Campus, Barcelona, Spain; ^8^Radiation Oncology Group, Vall d’Hebron Institute of Oncology (VHIO), Vall d’Hebron Barcelona Hospital Campus, Barcelona, Spain; ^9^Area of Clinical and Molecular Genetics, Vall d’Hebron Hospital Universitari, Vall d’Hebron Barcelona Hospital Campus, Barcelona, Spain

**Keywords:** radiotherapy—adverse effects, radiation-induced lymphocyte apoptosis (RILA), gene set enrichment analysis (GSEA), late skin toxicity, breast cancer

## Abstract

**Background:**

Radiation-induced late effects are a common cause of morbidity among cancer survivors. The biomarker with the best evidence as a predictive test of late reactions is the radiation-induced lymphocyte apoptosis (RILA) assay. We aimed to investigate the molecular basis underlying the distinctive RILA levels by using gene expression analysis in patients with and without late effects and in whom we had also first identified differences in RILA levels.

**Patients and Methods:**

Peripheral blood mononuclear cells of 10 patients with late severe skin complications and 10 patients without symptoms, selected from those receiving radiotherapy from 1993 to 2007, were mock-irradiated or irradiated with 8 Gy. The 48-h response was analyzed in parallel by RILA assay and gene expression profiling with Affymetrix microarrays. Irradiated and non-irradiated gene expression profiles were compared between both groups. Gene set enrichment analysis was performed to identify differentially expressed biological processes.

**Results:**

Although differentially expressed mRNAs did not reach a significant adjusted *p*-value between patients suffering and not suffering clinical toxicity, the enriched pathways indicated significant differences between the two groups, either in irradiated or non-irradiated cells. In basal conditions, the main differentially expressed pathways between the toxicity and non-toxicity groups were the transport of small molecules, interferon signaling, and transcription. After 8 Gy, the differences lay in pathways highly related to cell senescence like cell cycle/NF-κB, G-protein-coupled receptors, and interferon signaling.

**Conclusion:**

Patients at risk of developing late toxicity have a distinctive pathway signature driven by deregulation of immune and cell cycle pathways related to senescence, which in turn may underlie their low RILA phenotype.

## Introduction

Radiotherapy (RT) is commonly given to breast cancer (BC) patients after surgery, and it has been shown to be effective in reducing recurrence and lowering BC mortality (1). Studies have suggested that RT can benefit up to 83% of BC patients (2) and that breast-conserving surgery followed by whole-breast RT can provide local control and survival rates equivalent to mastectomy (3–5). However, early and late side effects caused by RT can negatively affect patients’ quality of life (6). Late or chronic effects are typically expressed after latent periods of months to years and are highly relevant as they tend to be irreversible or even progressive in severity (7, 8). Chronic toxicity in BC patients mainly comprises radiation-induced fibrosis, skin shrinkage or induration, atrophy, vascular damage, neural damage, lymphedema, myocardial infarction, secondary cancers, and a range of endocrine- and growth-related effects (6, 9). Treatment regimens are designed to ensure that the risk of severe late effects does not exceed 5%–10% (10). This means that a small fraction of radiosensitive patients imposes limits on the dose that can be given to the entire patient population, although the majority of patients could potentially tolerate a higher dose (11). A more effective approach would be adjusting the treatment according to patient and tumor radiosensitivity. In non-sensitive patients, a dose escalation could be considered, while in sensitive patients, interventions such as an alternative treatment (surgery), toxicity reduction RT techniques (e.g., partial breast RT), therapeutic (with radioprotectors or radiation mitigators), and the omission of postoperative RT in patients with a low risk for tumor recurrence might be considered to reduce their risk of side effects (11–13).

Although the relation to BC radiotoxicity of clinical factors such as smoking history, breast volume, additional boost delivery, and diabetes (14, 15) is well-founded, it is hypothesized that constitutional genetic factors are, in addition, key determinants of the wide interpersonal variability observed in clinical practice (16). Accordingly, over the past two decades, there has been an increasing interest in deciphering the germline genetic susceptibility underlying radiation-induced toxicity after RT, with studies focused on common genetic variants through candidate gene or genome-wide association studies (16–18). Likewise, different cellular and DNA markers after *in-vitro* irradiation of fibroblasts and lymphocytes have been tested to know if their response to radiation is related to clinical toxicity (19). Some investigations [including our work (20)] have shown that late toxicity due to RT is significantly associated with low levels of radiation-induced lymphocyte apoptosis (RILA) using 8 Gy of *in-vitro* irradiation and an incubation time of 48 h. The predictive value of the RILA assay in radiotherapy-induced fibrosis was validated within the PHRC2005 (NCT00893035) prospective multicenter French study (21). However, cell mechanisms determining a low level of RILA are not well known.

We aimed to shed light on the molecular pathogenesis of low RILA levels by characterizing RNA expression using microarrays on blood-derived cells for BC patients with and without fibrosis and/or telangiectasia induced by RT and in whom we had also demonstrated different levels in RILA (20). Our study reveals that gene expression differences between patients with and without late toxicity are driven by deregulation of immune and cell cycle pathways related to senescence, which may underlie the low RILA phenotype.

## Materials and Methods

### Patient Selection

Twenty BC patients were selected from those prospectively receiving RT between 1993 and 2007 in the Hospital de la Santa Creu i Sant Pau of Barcelona. Ten patients with fibrosis and/or telangiectasias were matched according to treatment, demographic, and tumor characteristics with 10 patients with no late reactions (**Tables 1**, **2**). Patients were given whole breast RT with conventional fractionation, 50 Gy, 2 Gy/fraction with 60 Co unit. An additional tumorectomy electron boost (9–16 MeV) from a linear accelerator was given, with doses ranging from 14 to 20 Gy. The dose was 20 Gy at 85% isodose. Planning was based on 2D simulation, including verification and quality assurance. The occurrence of acute and late effects of RT was monitored and documented by the physician during the standard patient follow-up for at least 6 years after RT, using the Radiation Therapy Oncology Group (RTOG) toxicity grading system (22) (**Table 1**). We determined RILA values in these patients showing that those with late RT toxicity exhibited reduced apoptosis, and these findings were published elsewhere (20). The ethical committee of the hospital approved the study and all women participating signed an informed consent.

**Table 1:** Acute and late toxicity in the analyzed breast cancer patients [adapted from (20)].

Group	Patient ID	Acute toxicity (evaluated ≤3 months after radiotherapy)	Late toxicity (evaluated >3 months up to at least 6 years after radiotherapy)	Year of end of radiotherapy	Last year of toxicity confirmation (years of follow-up)	Year of blood extraction for RILA/microarray studies
**Without late normal tissue reactions**						
	SP-13	Epithelitis	None	2003	2014 (11)	2010
	SP-18	None	None	2005	2014 (9)	2010
	SP-12	Erythema	None	2000	2014 (14)	2010
	SP-4	Epithelitis	None	1999	–	2009
	SP-16	Dermatitis	None	2004	2014 (10)	2010
	SP-7	None	None	2002	2011 (9)	2009
	SP-11	Erythema	None	1994	2011 (17)	2010
	SP-19	Erythema	None	2004	2013 (9)	2010
	SP-8	Epithelitis grade 2	None	1994	2013 (19)	2010
	SP-6	Epithelitis grade 3	None	2002	2012 (10)	2009
**With late normal tissue reactions**						
	SP-10	Erythema	Telangiectasia grade 1	2004	2013 (9)	2010
	SP-2	None	Telangiectasia grade 2 bilateral and fibrosis grade 2 in right breast	1994	2012 (18)	2009
	SP-1	Erythema and epithelitis	Telangiectasia grade 2 and fibrosis grade 2	2005	2013 (8)	2009
	SP-5	Erythema	Telangiectasia grade 1	2004	2011 (7)	2009
	SP-14	Erythema and moist epithelitis grade 3	Fibrosis grade 3	2007	2013 (6)	2010
	SP-17	Erythema	Fibrosis grade 3	2002	2011 (9)	2010
	SP-20	Erythema	Telangiectasia grade 2 and fibrosis grade 3	2002	2012 (10)	2010
	SP-9	Erythema and edema	Fibroadenoma grade 1	2006	2012 (6)	2010
	SP-3	Erythema	Telangiectasia grade 1	1998	2010 (12)	2009
	SP-15	Epithelitis grade 2 in each bilateral breast cancer	Telangiectasia and fibrosis grade 3 in each bilateral breast cancer	1993	2012 (19)	2010

**Table 2:** Clinical and demographic characteristics of the analyzed patients [adapted from (20)].

Characteristic	Non-toxicity group (n = 10)	Toxicity group (n = 10)
**Age at radiotherapy (mean ± SD)**	45.70 ± 10.01	52.80 ± 8.60^a^
**Age at study (mean ± SD)**	55.20 ± 8.01	61.60 ± 8.63^a^
**BMI (mean ± SD)**	25.42 ± 3.41 (*n* = 5)	26.90 ± 6.21^a^ (*n* = 4)
**Smoking status**		
**Non-smoker**	50% (*n* = 8)	70%^b^
**Current smoker**	12.5% (*n* = 8)	10%^b^
**Former smoker**	37.5% (*n* = 8)	20%^b^
**Histological type**		
**Invasive ductal**	70%	80%^b^
**Invasive lobular**	10%	0%^b^
***In situ***	10%	10%
**Other**	10% (mixed ductal and lobular)	10% (tubule-lobular carcinoma)
**Adjuvant treatment**		
**Neoadjuvant chemotherapy**	10%	0%^b^
**None**	10%	10%^b^
**Chemotherapy**	0%	10%^b, c^
**Hormone therapy**	20%	50%^b^
**Chemotherapy + hormone therapy**	60%	20%^b^
**Radiotherapy**		
**Basic irradiation dose**	50 Gy (*n* = 9), 46 Gy (*n* = 1)	50 Gy
**Boost dose**	14–20 Gy	16–20 Gy
**Total prescribed dose Gy (mean ± SD)**	65.80 ± 0.63^a^	67.00 ± 1.69^a^
**Boost with brachytherapy**	10%	10%
**Radiotherapy concomitant to chemotherapy**	30%	10%^b^

a No statistically significant differences between both groups: t-test.

b No statistically significant differences between frequencies of both groups: Yates’ corrected chi-square test.

c One of them was also treated with a monoclonal antibody as adjuvant therapy.

### Collection of Samples, Irradiation, and Incubation

Experiments for the RILA assay and microarray analysis were carried out in parallel with blood samples being collected between 2009 and 2010 (**Table 1**). For the microarray study, peripheral blood mononuclear cells (PBMCs) were isolated using Lymphoprep™ (STEMCELL Technologies, Vancouver, Canada) density gradient medium from whole blood. Two PBMC cultures were freshly set up for each of the 20 patients (with an average of 8.5 × 10^6^ cells in 20 ml of RPMI 1640 supplemented with 20% fetal bovine serum): i) one culture was treated with 8 Gy at room temperature at a dose rate of 3 Gy/min with 6 MV photon beam, and ii) one culture was the control sham-irradiated. Both cultures were then placed into a 37°C incubator with 5% CO_2_. The irradiation with 8 Gy was done at the same time for the RILA assay and microarray cultures.

### Microarray Assay

After 48 h of incubation (the same time as that used for the RILA assay), total RNA was isolated using TRIzol isolation reagent (Invitrogen, Thermo Fisher Scientific, Waltham, MS, USA); see **Supplementary Information** for more details on RNA purification. Gene expression was measured using an Affymetrix GeneChip™ Human Exon 1.0 ST Array (Affymetrix, Thermo Fisher Scientific, Santa Clara, CA, USA) for each RNA sample. One microgram of total RNA was used as starting material and ribosomal RNA was first removed using the RiboMinus Human/Mouse Transcriptome Isolation Kit (Invitrogen, Thermo Fisher Scientific, Waltham, MS, USA). Following the manufacturer’s instructions, treated RNA was then converted to cDNA and subsequently processed and labeled, to detect the transcripts by hybridization onto the Exon arrays. After hybridization, each array was washed and stained according to the standard Affymetrix protocol. Finally, the stained array was scanned using an Affymetrix GeneChip Scanner 3000, and the signals were processed by the GeneChip Operating System for each array.

### Statistical Analysis to Detect Differentially Expressed Genes

Raw expression values were obtained directly from .CEL files and were preprocessed using the Robust Multiarray Average method (23). Prior to any analysis, data were submitted to non-specific filtering to remove low signal genes and low variability genes. The selection of differentially expressed genes between conditions was based on a linear model analysis with empirical Bayes moderation of the variance estimates following the methodology developed by Smyth (24), by using the limma package from Bioconductor. To deal with multiple testing issues, *p*-values were adjusted to obtain strong control over the false discovery rate (FDR) using the Benjamini and Hochberg method (25).

The lists of genes differentially expressed were generated for the following comparisons:

- Basal expression: toxicity vs. non-toxicity patients in non-irradiated samples
- 8 Gy-induced expression^1^: toxicity vs. non-toxicity patients in irradiated samples
- Overall *in-vitro* radiation response: irradiated vs. non-irradiated samples, in all patients regardless of toxicity status and separately for each of the two groups

Heatmaps for both basal and each radiation response comparisons were also generated. R statistical software v4.03 and Bioconductor packages were used for all analyses.

### Gene Set Enrichment Analysis

Gene set enrichment analysis (GSEA) (version 3.0, Broad Institute http://software.broadinstitute.org/gsea/index.jsp) (26) was used to identify biological terms, pathways, and processes that were up- or downregulated within each pairwise comparison. The genes obtained from the array were ranked using the logFC from the differential expression results. This ranked list was then used to perform a GSEA using the Reactome gene sets database. The overlap between nodes was visualized by the generation of an enrichment map. The map was constructed with an FDR cutoff of <0.05, except for the comparison in basal conditions of patients with toxicity vs. non-toxicity, where the FDR cutoff was <0.25. R statistical software v4.03 and Bioconductor packages were used for the analysis. Finally, we searched the pathways in the Reactome website (www.reactome.org) to label the clusters to broadly account for functions of constitutive gene sets.

### Quantitative RT-PCR Validation

To validate the microarray expression data, reverse transcription-quantitative PCR (RT-qPCR) was performed using TaqMan probes (Applied Biosystems, Thermo Fisher Scientific, Waltham, MS, USA) targeting selected genes as well as reference controls to normalize data. For a more detailed RT-qPCR methodology, see **Supplementary Information**. Paired *t*-test was applied for statistical analysis, using GraphPad Prism 6 software. To deal with multiple testing issues, *p*-values were adjusted using the Benjamini and Hochberg method (25).

## Results

### Basal Expression: Toxicity vs. Non-Toxicity Patients in Non-Irradiated Samples

**Figure 1:**
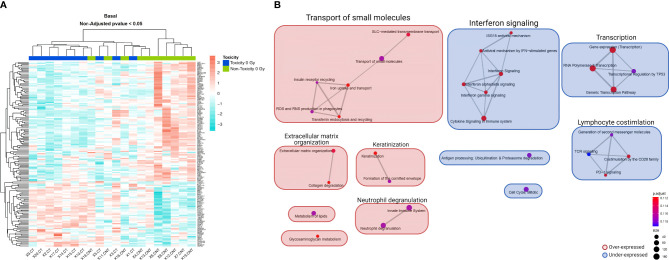
Heatmap and GSEA of basal non-irradiated cells comparing patients with late skin toxicity to normal responders. **(A)** A different expression pattern between patients with toxicity (dark blue) and without toxicity (green) of baseline expressed genes with non-adjusted p-value < 0.05 is suggested by hierarchical clustering classification of patients (columns). **(B)** The enrichment map allows the visualization of the general expression landscape of the toxicity group compared with the non-toxicity group in basal conditions. The key is shown in the bottom right of the figure. The map is constructed with an FDR cutoff of <0.25. Each node corresponds to a gene set from the GSEA; its size represents the number of genes in the gene set and its color the *p*-value. The thickness of the lines connecting the nodes is proportional to the number of genes that overlap in the gene set. Clustering among the various gene sets is visualized with boxes over the nodes. Those sets that are upregulated in patients with toxicity are shown in red and those downregulated are shown in blue. CT, control non-irradiated sample in toxicity patients; CNT, control non-irradiated sample in non-toxicity patients; GSEA, gene set enrichment analysis.

In basal non-irradiated cultures, no differentially expressed genes with adjusted *p <*0.05 were found between the 10 patients with late skin toxicity and the 10 without (**Supplementary Table 1**). The data visualization through heatmaps showed that mRNA expression clusters 13 of the 20 non-irradiated PBMC samples into two distinct groups depending on whether they are BC patients with (left) or without (right) RT-induced late toxicity, except for patient SP-18 (**Figure 1A**). A third group (middle) of seven patients did not discriminate between toxicity statuses.

We performed a GSEA with the whole list of ranked genes, since the analysis at the pathway level may be more biologically meaningful than considering individual genes, even if the changes in gene expression levels are minor or go undetected. In this case, 16 marginally significant enriched pathways were observed with an adjusted *p* = 0.1117 (**Supplementary Table 2**). The enrichment map shows that the two largest differentially expressed gene clusters in toxicity patients compared with normal responders are an overexpressed transport of small molecules and an underexpressed interferon signaling, both with six nodes, followed by an underexpression of transcription and lymphocyte costimulation clusters (**Figure 1B**).

### 8 Gy-Induced Expression: Toxicity vs. Non-Toxicity Patients in Irradiated Samples

**Figure 2:**
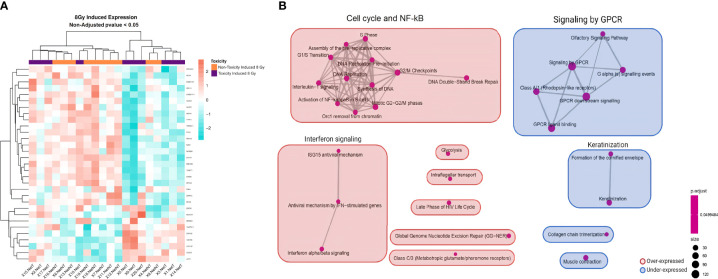
Heatmap and GSEA of 8 Gy-induced expression comparing patients with late skin toxicity to normal responders. **(A)** A different expression pattern between patients without toxicity (orange) from those with toxicity (purple) of 8 Gy-induced expressed genes with non-adjusted *p*-value <0.05 is suggested by hierarchical clustering classification of patients (columns). **(B)** The enrichment map allows the visualization of the general expression landscape induced by 8 Gy of the toxicity group compared with the non-toxicity group. The key is shown in the bottom right of the figure. The map is constructed with an FDR cutoff of <0.05. Each node corresponds to a gene set from the GSEA; its size represents the number of genes in the gene set and its color the *p*-value. The thickness of the lines connecting the nodes is proportional to the number of genes that overlap in the gene set. Clustering among the various gene sets is visualized with boxes over the nodes. Those sets that are upregulated in patients with toxicity are shown in red and those downregulated are shown in blue. NetT, 8 Gy-induced expression in toxicity patients; NetNT, 8 Gy-induced expression in non-toxicity patients; GSEA, gene set enrichment analysis.

No differentially expressed genes were found with an adjusted *p <*0.05 when comparing irradiation-induced expression levels between the 10 patients with late skin toxicity and the 10 without (**Supplementary Table 3**). The heatmap of the 8 Gy-induced expression showed that most of the patients were grouped according to their toxicity status (**Figure 2A**). After clustering the list of genes in pathways, significant differences could be observed (**Supplementary Table 4**). The main overrepresented gene sets in late toxicity patients were an upregulated cell cycle/NF-κB cluster and interferon signaling pathway together with underexpressed signaling by the G-protein-coupled receptor (GPCR) cluster (**Figure 2B**).

### Overall *In-Vitro* Radiation Response: Irradiated vs. Non-Irradiated Samples

**Figure 3:**
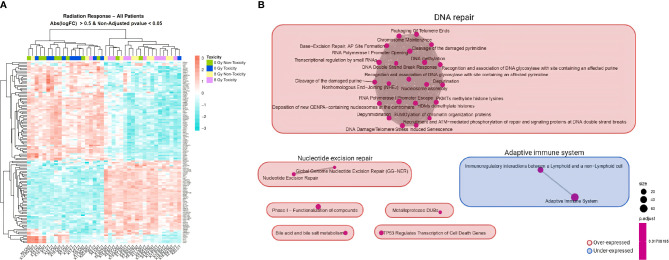
Heatmap and GSEA of overall *in-vitro* radiation response comparing irradiated to non-irradiated samples in all patients. **(A)** A clear different expression pattern is observed between non-irradiated (non-toxicity in green and toxicity in dark blue) and 8 Gy-irradiated (non-toxicity in yellow and toxicity in pink) PBMC samples. Hierarchical clustering of the differentially expressed genes with non-adjusted *p <*0.05 and absolute logFC >0.5 almost perfectly separates the two experimental conditions. **(B)** The enrichment map allows the visualization of the general expression landscape of the irradiated compared with the non-irradiated PBMCs. The key is shown in the bottom right of the figure. The map is constructed with an FDR cutoff of <0.05. Each node corresponds to a gene set from the GSEA; its size represents the number of genes in the gene set and its color the *p*-value. The thickness of the lines connecting the nodes is proportional to the number of genes that overlap in the gene set. Clustering among the various gene sets is visualized with boxes over the nodes. Those sets that are upregulated in irradiated samples are shown in red and those downregulated are shown in blue. Abs, absolute; CNT, control non-irradiated sample in non-toxicity patients; 8 Gy NT, 8 Gy-irradiated culture in non-toxicity patients; CT, control non-irradiated sample in toxicity patients; 8 Gy T, 8 Gy-irradiated culture in toxicity patients; PBMCs, peripheral blood mononuclear cells; GSEA, gene set enrichment analysis.

Microarray analysis of the 20 donors regardless of their toxicity status indicated that *in-vitro* irradiation led to a differential expression of 753 genes with an adjusted *p <*0.05 (**Supplementary Table 5**). RT-qPCR results validated the overexpression of *APOBEC3H* (adjusted *p* = 0.0002) (**Supplementary Figure 1**). A hierarchical clustering analysis depicted in a heatmap showed that the mRNA expression of most PBMC samples clustered into two distinct groups (non-irradiated or irradiated), except for patients SP-11, SP-12, and SP-20 (**Figure 3A**). After clustering the differentially expressed genes between non-irradiated and irradiated cells of the 20 patients (**Supplementary Table 6**), the enrichment map showed that the main significant upregulated pathway was DNA repair with 22 nodes followed by nucleotide excision repair and downregulated adaptive immune system with two nodes each (**Figure 3B**).

**Figure 4:**
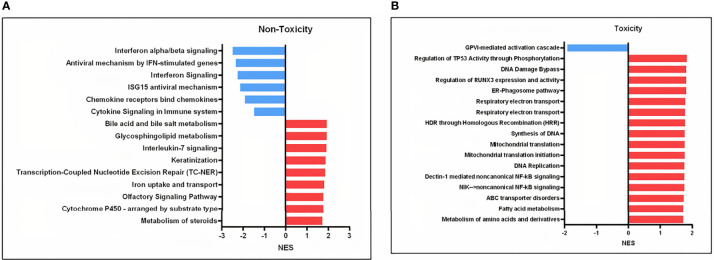
Involved pathways in cell response to 8 Gy comparing irradiated to non-irradiated samples in the toxicity and non-toxicity patients. **(A)** Gene sets exclusively downregulated (blue) and upregulated (red) in the non-toxicity group after 8 Gy. **(B)** Gene sets exclusively downregulated (blue) and upregulated (red) in the toxicity group after 8 Gy. NES, normalized enrichment score.

To characterize the expression profile after *in-vitro* irradiation for each group of patients, we generated separate lists of differentially expressed genes according to toxicity group (**Supplementary Tables 7, 8**). The RT-qPCR showed that *APOBEC3H* was significantly upregulated in both groups of patients (**Supplementary Figure 2**). Interestingly, the heatmaps generated from each group of patients indicated that the clustering of expressed genes in non-irradiated and irradiated cells was different between normal responders and sensitive patients (**Supplementary Figure 3**). In line with these results, the map of significant enriched pathways was different between the two groups, with patients without side effects having two clusters of pathways downregulated not present in the toxicity group (**Supplementary Figure 4**). Looking at the common single pathways that are differentially expressed after irradiation in the toxicity and non-toxicity patients, 107 pathways were found (**Supplementary Tables 9, 10**). On the contrary, 15 and 17 pathways were unique to the non-toxicity and toxicity groups, respectively, mainly related to NF-κB and interferon signaling (**Figures 4A, B**).

## Discussion

The purpose of this study was to determine whether late clinical toxicity after BC RT and a low level of RILA is associated with an altered transcriptional profile of PBMCs. The analyzed patients were well defined by their toxicity and RILA status and were selected from a larger cohort treated with RT between 1993 and 2007 with an average follow-up of 11 years (**Tables 1**, **2**) (20). Our comparison of the resulting microarray data for patients with late toxicity vs. patients without shows that, after correcting for multiple testing, no individual gene met the threshold for statistical significance. This may be because expression differences are modest relative to the possible noise resulting from a heterogeneous cell fraction included in PBMCs and to the intrinsic heterogeneity in patient samples. In contrast, our pathway analysis indicates a statistically significant enrichment of certain biological mechanisms that would contribute to toxicity and low RILA levels.

Other microarray studies have been conducted in recent years to characterize baseline or irradiated expression profiles using lymphocytes, whole blood, or skin fibroblasts from cancer patients with adverse reactions in normal tissue after RT, and all of these studies support that gene expression profiles might discriminate patients at risk [reviewed in (19, 27)]. In some cases, patient classification was improved by considering the differential expression of functionally related genes belonging to the same pathway, which might be more significant than the activity of individual genes and can also lead to a more relevant biological interpretation of the results (27).

Regarding BC studies, Henríquez Hernández et al. (18) identified 26 genes whose basal expression in lymphocytes segregated patients suffering severe late skin toxicity from patients who did not. Their enrichment pathway analysis shows similar pathways identified to those we have seen in our study for basal conditions like T-cell receptor signaling, transcription, and lipid/cholesterol metabolism (18) (**Figure 1B**). Landmark-Høyvik et al. (28) compared the whole blood basal gene expression of BC patients that developed fibrosis after RT to those that did not, and they found differentially expressed pathways such as transcription and intercellular transport/localization (28), which were also found deregulated in our study in basal conditions (**Figure 1B**). In contrast, we found other differentially expressed pathways not reported in these last two studies such as interferon signaling, keratinization, and neutrophil degranulation (**Figure 1B**). Rødningen et al. found 60 differentially expressed genes after irradiation in fibroblast cultures from 31 BC patients with variable risk of radiation-induced fibrosis which were involved in processes like apoptosis, extracellular matrix remodeling/cell adhesion, proliferation, and ROS scavenging (29). As opposed to their study, we saw different responses between the toxicity and non-toxicity groups after irradiation in the cell cycle/NF-κB cluster, signaling by GPCR, and interferon (**Figure 2B**). Variations in cell type, microarray platform, *in-vitro* irradiation protocol, RT protocol, and statistical strategy could influence the final outcome and explain why different molecular pathways are highlighted between different reports.

Our GSEA highlights a role in late toxicity for interferon signaling both in basal and irradiated PBMCs, a finding not previously reported in similar studies (**Figures 1B**, **2B**). Interferons are key signaling molecules for innate and adaptive immunity also implicated in cell senescence with a complex interplay (30). It has been described that there is an upregulation of interferon signaling in senescent cells and that the DNA damage process engages cell senescence through interferon signaling (31). Moreover, it is known that ionizing radiation can induce senescence in normal cells, which promotes normal tissue fibrosis and organ dysfunction [reviewed in (32, 33)]. A growing body of evidence supports that senescence may be at the origin of radiation-induced pulmonary and skin fibrosis (31).

Likewise, we also found an overrepresentation of upregulated NF-κB nodes in the group of patients with late skin toxicity in irradiated conditions (**Figures 2B**, **4B**). After radiation-induced DNA damage, ATM protein activates DNA damage response pathways which lead to the stimulation of NF-κB that induces senescence-associated secretory phenotype (SASP) pro-inflammatory expression including IL-1α/β, IL-6, TGF-β, TNF-α, and fibroblast growth factor, among others [reviewed in (31, 32)].

In the irradiated PBMCs, we also observed a significant overrepresentation of a downregulated GPCR signaling pathway in patients suffering toxicity (**Figure 2B**). GPCRs are involved in signal transduction and play important roles in nearly every physiological process including DNA damage response and cell senescence (34, 35). Interestingly, a functional enrichment analysis of genes to detect loci for acute post-RT pain in 1,112 BC patients showed that one of the most significant enriched biological processes was olfactory receptor activity, which are members of GPCR signaling (36).

Thus, the co-occurrence of our findings in irradiated PBMC of patients with toxicity of an upregulated NF-κB and interferon signaling process as well as a downregulated GPCR pathway (all known to be connected with cell senescence) might not be accidental and suggest their importance for RT-induced late toxicity phenotype.

Our cohort of patients with late toxicities is characterized by a low level of RILA after 48 h post-irradiation with 8 Gy (20). A time course RNA expression analysis adding for instance a 24-h post-irradiation time point would have led to a more comprehensive profile. However, previous published evidence showed that the correlation between low RILA values and patients who did develop radiation-induced toxicity is seen both at 24 and 48 h post-irradiation (20, 37–40). Likewise, other reports point to an increase in radiation-induced apoptosis with radiation dose and time of incubation after *in-vitro* irradiation in both sensitive and non-sensitive individuals (41, 42), which indicates that the apoptosis levels seen at 48 h would not be preceded by higher apoptosis at previous time points.

Our results of an overrepresentation of processes highly involved in senescence suggest that it would be interesting to further investigate its implication for low RILA levels shown by patients with late toxicity. However, it should be considered that we quantified a bulk gene expression derived from a variety of cells included in the PBMC fraction (43), while a correlation of late toxicity with decreased values of RILA has been shown only in T and B lymphocytes (20, 44, 45). In humans, the frequency of the fraction of comprised cells in PBMC populations varies across individuals, but typically, lymphocytes are in the range of 70%–90%, monocytes from 10% to 20%, while dendritic cells are rare, accounting for only 1%–2% (43). The frequencies of cell types within the lymphocyte population include 70%–85% CD3^+^ T cells, 5%–10% B cells, and 5%–20% NK cells (43). In consequence, the largest fraction of PBMCs corresponds to T lymphocytes, and therefore, the gene expression profile observed might be a reflection of this majority cell fraction.

When studying the effect of radiation in all patients, we saw that irradiation of PBMCs led to the induction of Paul and Amundson’s consensus list of radioresponsive genes (46–48) such as *CDKN1A*, *GADD45A*, *DDB2*, *XPC*, and *PCNA* (**Supplementary Table 11**). As expected, the biggest cluster of differentially expressed pathways was DNA repair (**Figure 3B**), which is consistent with previous studies (48, 49). Identifying this radiation signature was reassuring and supported microarray experiment conditions and derived findings.

In conclusion, although the results indicate that patients with and without toxicity share common cell responses, the enriched pathways are different between both groups for basal and *in-vitro* irradiation conditions. The resulting pathways associated with late toxicity (such as interferon signaling, NF-κB, and signaling by GPCR) support a key role of cell senescence in RT-induced late toxicity and low RILA phenotype.

## Data Availability Statement

Microarray data for this study can be found in NCBI Gene Expression Omnibus (https://www.ncbi.nlm.nih.gov/geo/) under the accession number GSE178708.

## Ethics Statement

The studies involving human participants were reviewed and approved by the Ethical Committee for Clinical Research Santa Creu i Sant Pau Hospital. The patients/participants provided their written informed consent to participate in this study.

## Author Contributions

EA-F, MF-R, CA, AS, OD, and SG-E participated in the conception of the study. EA-F, MF-R, CA, TR, DF, AS, ASP, and SG-E contributed to the data collection. Statistical analysis and interpretation of data were executed by EA-F, RG, ASP, and SG-E. OD and SG-E contributed to supervision. SG-E contributed to the study management. The first draft of the manuscript was written by EA-F. EA-F, OD, and SG-E reviewed and edited the manuscript. JG and SG-E acquired funding and resources. All authors read the manuscript and approved the final version.

## Funding

This work has been funded by the Instituto de Salud Carlos III (ISCIII), an initiative of the Spanish Ministry of Economy and Innovation partially supported by the European Regional Development FEDER Funds, Grant/Award Number: PI05/2181. SG-E was supported by the ISCIII Miguel Servet II Program (CP16/00034). EA-F was supported by the ERAPerMed JTC2018, grant numbers: ERAPERMED2018-244 and SLT011/18/00005.

## Conflict of Interest

The authors declare that the research was conducted in the absence of any commercial or financial relationships that could be construed as a potential conflict of interest.

## Publisher’s Note

All claims expressed in this article are solely those of the authors and do not necessarily represent those of their affiliated organizations, or those of the publisher, the editors and the reviewers. Any product that may be evaluated in this article, or claim that may be made by its manufacturer, is not guaranteed or endorsed by the publisher.

## Footnotes

1 Net values were obtained by subtracting baseline RNA expression levels from those obtained after irradiation.
