# Supplementary material for: Cell Senescence-Related Pathways Are Enriched in Breast Cancer Patients With Late Toxicity After Radiotherapy and Low Radiation-Induced Lymphocyte Apoptosis
Source: Front Oncol. 2022 May 24;12:825703. doi: 10.3389/fonc.2022.825703 (PMC9170959; doi:10.3389/fonc.2022.825703)
Supplement: Supplementary file 1 [file DataSheet_1.docx]

Supplementary Material

# Supplementary Information

## Materials and Methods

### Isolation and Purification of RNA

After 48 h of incubation (the same time as that used for RILA), total RNA was isolated using TRIzol Isolation Reagent (Invitrogen, Life Technologies). RNA was purified using Rneasy Minikit (QIAGEN) and DNA traces were digested with the RNase-Free DNase Set (QIAGEN). RNA concentrations were determined with a NanoDrop ND-1000 spectrophotometer (Thermo Fisher Scientific). RNA purity was assessed with the 260/280 and 260/230 nm ratios and RNA integrity was checked by gel electrophoresis in ethidium bromide 2% e-gels (Invitrogen, Thermo Fisher Scientific). RNA was frozen and kept at -80ºC until the time of usage.

### Quantitative RT-PCR validation

To validate the microarray expression data using reverse transcription-quantitative PCR (RT-qPCR), we selected two genes (PDK1 and APOBEC3H) of the significantly differentially expressed gene lists due to the limited quantity of RNA. We used predesigned human-specific primers and TaqMan probes (Applied Biosystem, Thermo Fisher Scientific): PDK1 (Hs01561847_m1) and APOBEC3H (Hs00962174_m1). ACTB (Hs03023880_g1), HRPT1 (Hs99999909_m1) and GAPDH (Hs02758991_g1) were used as reference controls to normalize data.

There was not enough RNA from the non-irradiated SP-15 and the irradiated SP-13 samples and finally 38 samples were analysed (19 irradiated and 19 non-irradiated). A total of four 384-well plates were set up. A calibrator non-irradiated RNA sample was added in both plates to allow comparison between plates. To generate cDNA, 100 ng of total RNA were reverse transcribed using PrimeScript Reverse Transcription Reagent Kit (Takara) that combines oligo-dT and random primers. Three reverse transcription replicates were done per each sample. qPCR assays were performed for each of the three reverse transcription replicates using for each sample 1µl of the 20x TaqMan gene expression assay, 10µl of 2x TaqMan Universal Master Mix II (Applied Biosystems), 8 µl of water and 1 µl of the cDNA (10ng) previously generated. Assays were run in a 7900HT Fast Real-Time PCR System (Applied Biosystems) and QuantStudio 6 Real-Time PCR System using default cycle conditions.

# Supplementary Figures


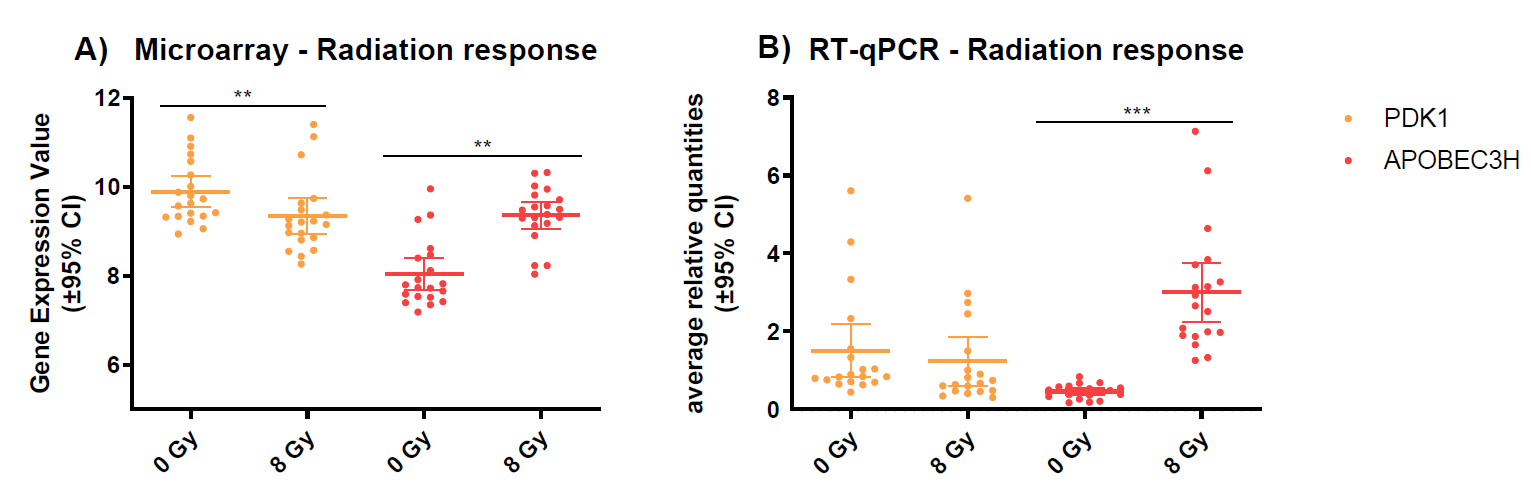


**Supplementary Figure 1.** Boxplots comparing microarray outcome with RT-qPCR results for 8 Gy of in vitro irradiation response in all 20 patients. A) Microarray data is shown on the right, **adjusted p-value<0.01 and B) RT-qPCR on the left, paired T-test ***adjusted p-value<0.001; PDK1 (orange) and APOBEC3H (red) values in irradiated and non-irradiated samples. Abbreviations: CI: confidence intervals; RT-qPCR: Real-time quantitative PCR.


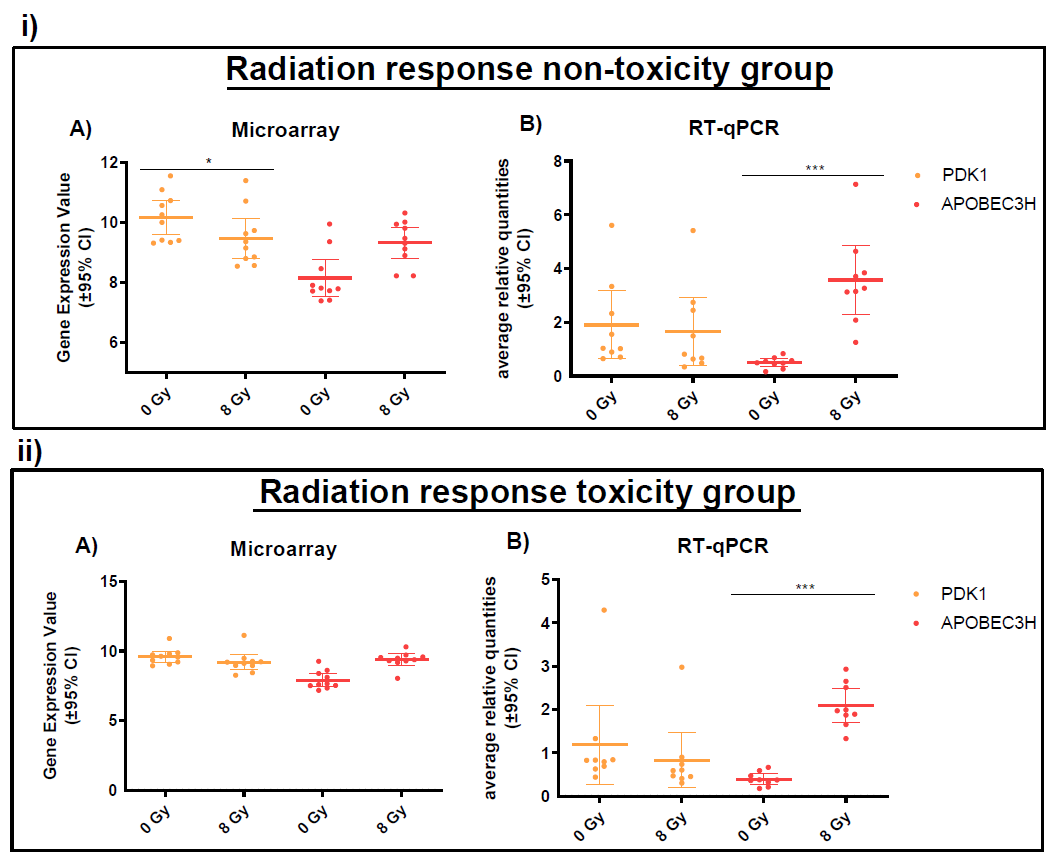


**Supplementary Figure 2.** Boxplots comparing microarray outcome with RT-qPCR results for 8 Gy response by groups: i) Non-toxicity group and ii) Toxicity group. A) Microarray data is shown on the right, *adjusted p-value<0.05 and B) RT-qPCR on the left, paired T-test **p-value<0.01 and ***p-value<0.001; PDK1 (orange) and APOBEC3H (red) expression values in irradiated and non-irradiated samples. Abbreviations: CI: confidence intervals; RT-qPCR: Real-time quantitative PCR.


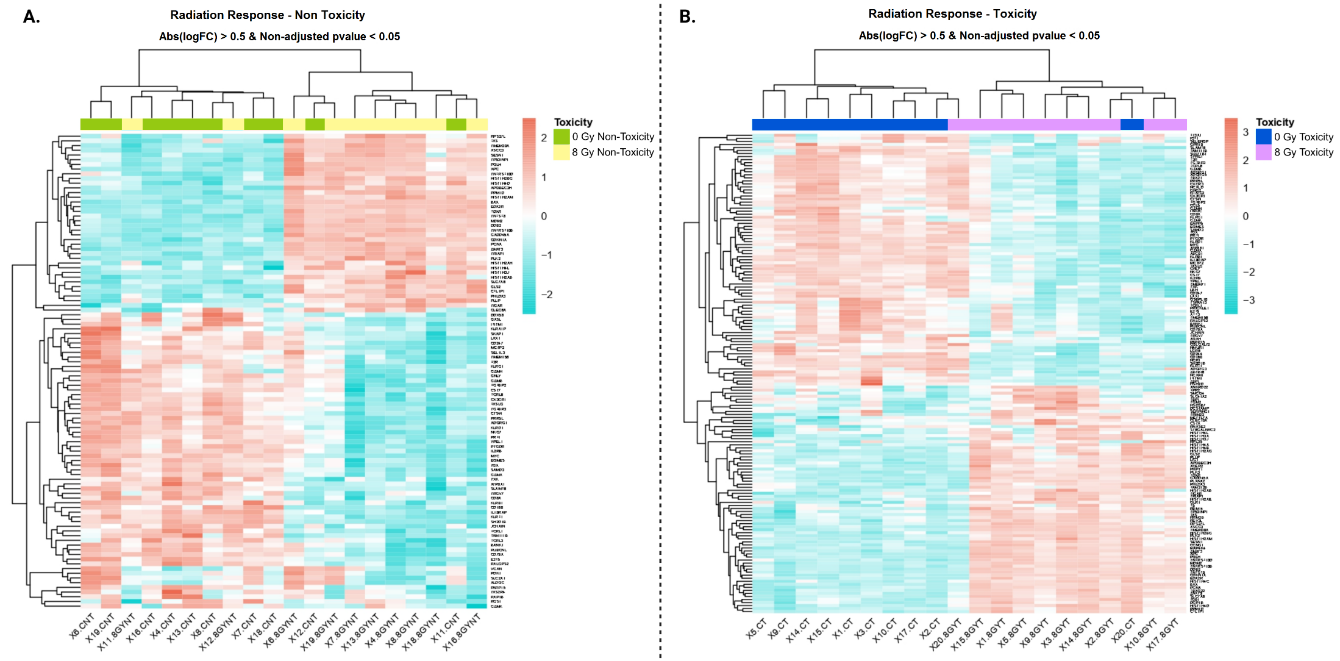


**Supplementary Figure 3**. Heatmap of 8 Gy of in vitro irradiation response by comparing irradiated to non-irradiated samples in toxicity and non-toxicity groups. Different expression pattern of selected genes with non-adjusted p-value < 0.05 and absolute logFC>0.5 is observed by hierarchical clustering classification of patients (columns), between irradiated samples and non-irradiated samples in A. without toxicity non-irradiated (green) and irradiated (yellow) and B. with toxicity non-irradiated (dark blue) and irradiated (pink). Abbreviations: Abs: absolute.


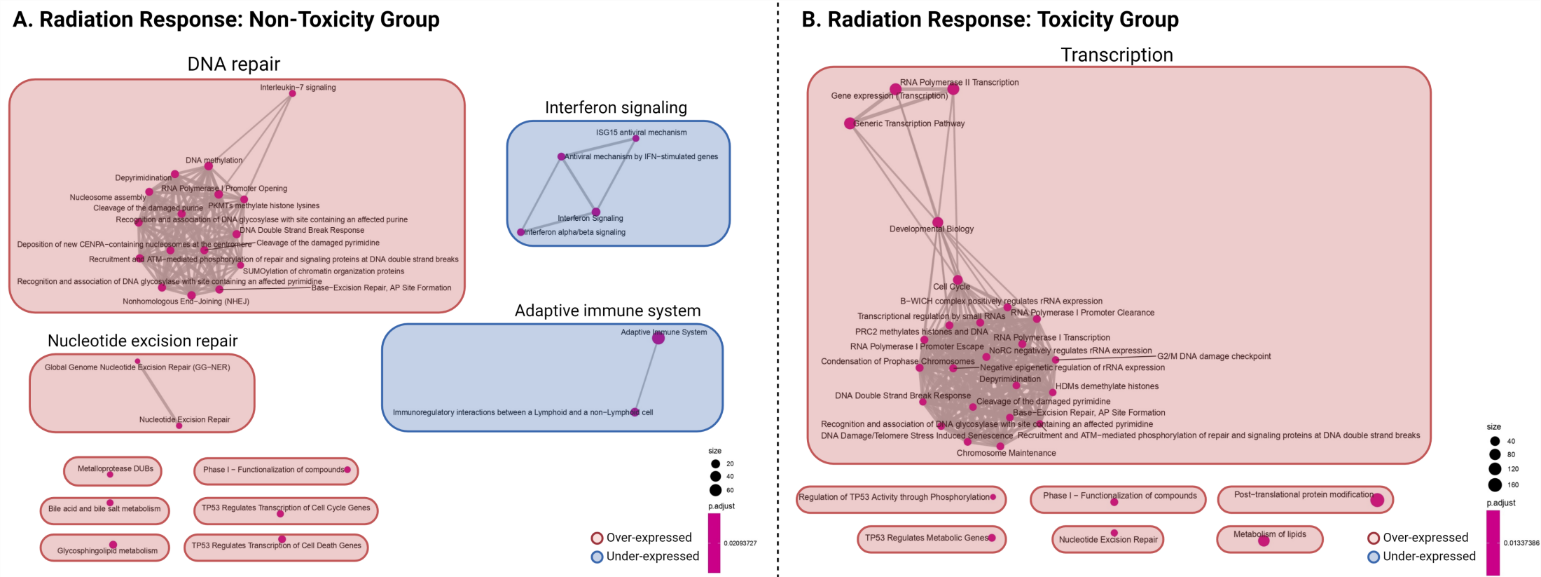


**Supplementary Figure 4.** GSEA of 8 Gy in vitro irradiation response comparing irradiated to non-irradiated samples by toxicity and non-toxicity groups. The enrichment map allows the visualization of the general expression landscape of the irradiated compared to the non-irradiated PBMCs for patients A. without toxicity and B. with toxicity. The key is shown in the bottom right of the figure. The map is constructed with an FDR cut-off of <0.05. Each node corresponds to a gene set from the GSEA; its size represents the number of genes in the gene set and its color the p-value. The thickness of the lines connecting the nodes is proportional to the number of genes that overlap in the gene set. Clustering among the various gene sets is visualized with circles over the nodes. Those sets that are upregulated in irradiated samples are shown in red and those downregulated are shown in blue. Abbreviations: PBMCs: Peripheral blood mononuclear cells; GSEA Gene set enrichment analysis.
